# Substrate Specificity of B12‐Depedent Ribonucleotide Reductases: Biotechnology and Metabolic Implications

**DOI:** 10.1002/cbic.202500344

**Published:** 2026-06-15

**Authors:** Lobna Eltoukhy, Christoph Loderer

**Affiliations:** ^1^ Nucleotide Biotechnology Group Chair of Molecular Biotechnology Dresden Germany

**Keywords:** allosteric regulation, biocatalysis, non‐canonical nucleotides, non‐natural nucleic acids, ribonucleotide reductases

## Abstract

Ribonucleotide reductases (RNRs) catalyze one of the central biochemical reactions, giving rise to deoxyribonucleotides, the building blocks of DNA. Due to their importance in cellular metabolism, this class of enzymes has been extensively studied over five decades. One aspect that has been neglected so far is the substrate specificity in terms of noncanonical nucleotides. While some of these compounds are physiologically relevant, many non‐natural nucleotides are important in medical science, biotechnology and synthetic biology. In this study, we investigated the substrate specificity of two thermostable RNRs for a broad range of natural and non‐natural nucleotides, in order to define the substrate promiscuity of this class of enzymes. Both enzymes were capable of converting all canonical nucleotides and a variety of other nucleotides. Generally, the enzymes were more likely to convert substrates with modifications of already existing functional groups of the nucleobase core structure. Our results show the potential and limitations for the biotechnological application of RNRs. In addition, they improve our understanding of the natural nucleotide metabolism in dealing with naturally occurring nucleotide analogues.

## Graphical abstract

Ribonucleotide reductases (RNRs) are valuable biocatalysts for the biosynthesis of non‐natural deoxyribonucleotide, relevant in fields like medical science and synthetic biology. In this study, we explore the substrate spectrum of two thermostable RNRs and discuss their implications for nucleotide metabolism and biocatalytical applications.

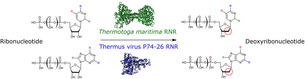

## 1 Introduction

Few molecules in biology match the importance of nucleotides for the living world. They play a central role in energy metabolism, catalysis, regulation, and the storage and propagation of information. In the form of their polymer DNA, 2′‐deoxyribonucleotides in particular, are responsible for coding every single bit of genetic information in all organisms known to biology. This grants deoxyribonucleotides an important role in fields ranging from medical science over biotechnology to synthetic biology.

In medical science, analogues of deoxyribonucleotides often are the pharmaceutically active compound of drugs. One remarkable example is the anti‐cancer drug cladribine (Mavenclad), which has recently been approved for the treatment of multiple sclerosis [1, 2]. In synthetic biology, the augmentation of the genetic code with additional non‐natural base pairs is a prime target. Here, the aim is to create orthogonal genetic systems or increase the already high information density of DNA molecules [3, 4].

For all their importance, the synthesis of (deoxy‐)ribonucleotides is a difficult endeavor. Chemical synthesis is a difficult multistep procedure, hampered by limitations in selectivity and yields [5, 6]. To overcome these difficulties, biocatalytic alternatives are being investigated extensively [7]. Generally, biocatalytic strategies for the production of ribonucleotides start with coupling the desired nucleobase on a ribose moiety, giving rise to either the respective nucleoside or nucleoside monophosphate. This is performed either by phosphoribosyl transferases [8, 9, 10] or purine nucleoside phosphorylases [11, 12, 13]. This is followed by kinase catalyzed phosphorylation steps, to give rise to the desired nucleotide, which is in most cases the nucleoside triphosphate. For these steps, different nucleotide kinases [14, 15] or polyphosphate kinases can be applied [16, 17]. In order to obtain a deoxyribonucleotide, there are different modifications of the above‐mentioned strategies.

Deoxyribose may be applied from the start of the cascade, or the nucleobase on an existing natural deoxyribonucleoside may be exchanged for the desired non‐natural nucleobase, both giving rise to modified deoxyribonucleosides [14, 18]. Alternatively, the nucleoside triphosphate is produced as described above and is then reduced to the corresponding deoxyribonucleoside triphosphate [10, 17].

**FIGURE 1:**
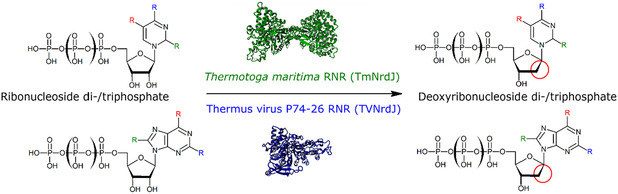
RNR reaction. Reduction of canonical and noncanonical ribonucleoside di‐ or triphosphates to the corresponding deoxyribonucleotides. The reactions are catalyzed by the dimeric nucleoside diphosphate specific TmNrdJ from *Thermotoga maritima* (PDB‐ID: 1XJE) and the monomeric nucleoside triphosphate specific TVNrdJ from the Thermus virus P74−26 (Homology model). Exemplary sites of modifications are labeled in red, blue, and green. The red circles indicate the reduced carbon.

In nature, as in published artificial cascades, this reaction is catalyzed by ribonucleotide reductases (RNR) (Figure 1). RNRs are ubiquitous in all known living organisms and some viruses and constitute the only known metabolic pathway for the de novo biosynthesis of deoxyribonucleotides [19, 20, 21].

All known RNRs share a common fold and a thiyl radical based reaction mechanism. However, they differ in their mechanism to generate this radical and as a consequence, they also differ in their relation to oxygen. Class I RNRs generate the radical via the cleavage of molecular oxygen in a di‐metal center or a DOPA dependent mechanism and ultimately depend on oxygen [22, 23, 24]. Class III RNRs generate the radical via S‐Adenosyl‐L‐methionine and store the radical on a glycine residue, which makes them oxygen sensitive [25, 26]. Class II RNRs generate the radical via cofactor B12, which makes them indifferent to the presence or absence of oxygen [27, 28].

In terms of substrate specificity, all class I and some class II RNRs reduce nucleoside diphosphate substrates, while the rest of class II and class III enzymes reduce nucleoside triphosphates [20, 21, 29]. In general, RNRs are capable of the reduction of the four canonical nucleotides but they are subject to a complex mechanism of allosteric regulation. In a mechanism for overall activity regulation, binding of mainly ATP or dATP to a separate ATP‐cone domain modifies the activity of the enzyme towards all potential substrates [30]. In a separate specificity regulation, the binding of one dNTP or ATP (effector) in a specificity site modifies the active site to preferentially reduce one NDP or NTP substrate [27, 30, 31, 32]. Binding of a dNTP in the specificity site modifies a loop structure that is part of the active site, responsible for binding of the base moiety of the substrate. For instance, the binding of dATP in the specificity site activates an RNR for the conversion of CDP. These substrate/effector pairs (ADP/dGTP, CDP/dATP, and GDP/dTTP), also called cognate pairs are well conserved within most RNRs. The in vivo function of this mechanism is to balance the nucleotide pools in order to reduce the mutation rate in DNA replication [31].

While the substrate spectrum and allosteric regulation of RNRs for their natural substrates is well‐described, little is known about their capability to convert noncanonical or non‐natural nucleotides. A study from the late 1970ies demonstrates the conversion of some non‐natural nucleotides by the class II RNR from *Lactobacillus leichmannii* [33]. Recently, the thermostable RNR from Thermus virus P74−26 has been applied for the biosynthesis of the noncanonical deoxyribonucleotides cladribine triphosphate and 6‐mercaptoguanosine triphosphate [10, 17].

In this study, we explore the substrate spectrum and allosteric regulation of two thermostable class II RNRs with differing phosphate specificity for the conversion of noncanonical and non‐natural nucleotides. We discuss the implications of our data for the potential and limitation for biotechnological applications as well as for the cellular nucleotide metabolism.

## 2 Results and Discussion

### 2.1 Enzyme Selection and Characterization

For a potential biotechnological application, enzymes need to meet a long list of requirements such as availability, stability, and applicability. Due to their indifference towards oxygen and the presence of enzymes with di‐ and triphosphate specificity, we selected class II RNRs. As a proxy for process stability, we favored class II RNRs originating from thermophilic organisms. Therefore, we selected the class II RNRs from Thermus virus P74−26 (TVNrdJ) [34, 35] and *T. maritima* (TmNrdJ) [27, 32] as model enzymes, with tri‐ and diphosphate specificity, respectively.

**FIGURE 2:**
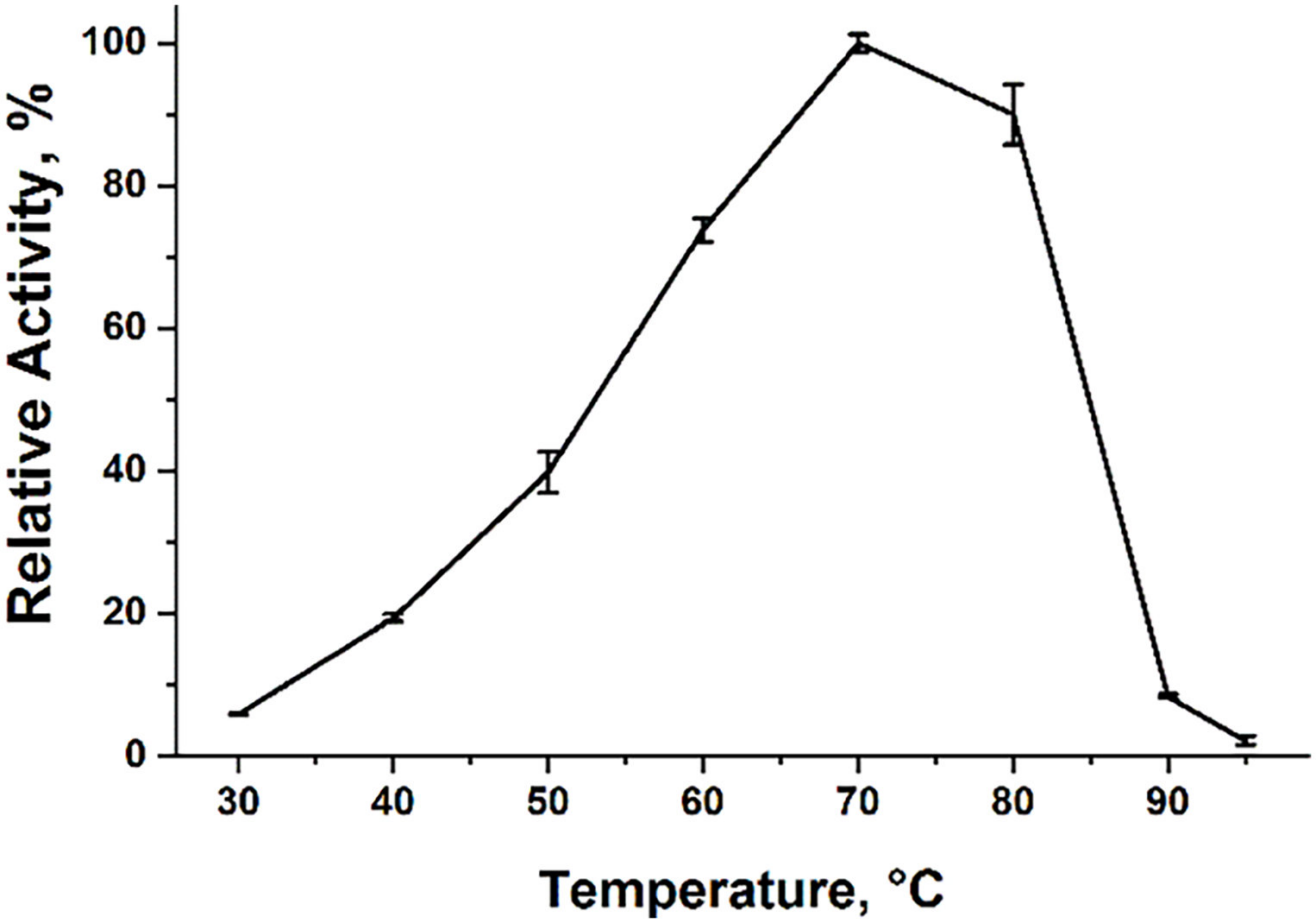
TmNrdJ reaction temperature. Temperature dependence of the TmNrdJ for the reduction of the substrate, CDP in the presence of the allosteric effector dTTP. The error bars indicate the standard deviation from three independent experiments.

Both enzymes were produced by recombinant expression in *E. coli* and purified by nickel affinity chromatography (Figure S1). The production yielded 16.2 mg L^−1^ for TmNrdJ and 49.2 mg L^−1^ for TVNrdJm. In order to establish reaction conditions for the characterization of the substrate spectrum, we investigated the temperature dependence of both enzymes. The temperature profile of the TVNrdJ was previously published, with a maximum reaction velocities at 60°C–70°C [34]. The temperature dependence was measured for the TmNrdJ under similar conditions in this study (Figure 2). The enzyme showed highest activity between 60°C and 80°C. For all further activity assays, 60°C was chosen as reaction temperature.

### 2.2 RNR Substrate Specificity

**FIGURE 3:**
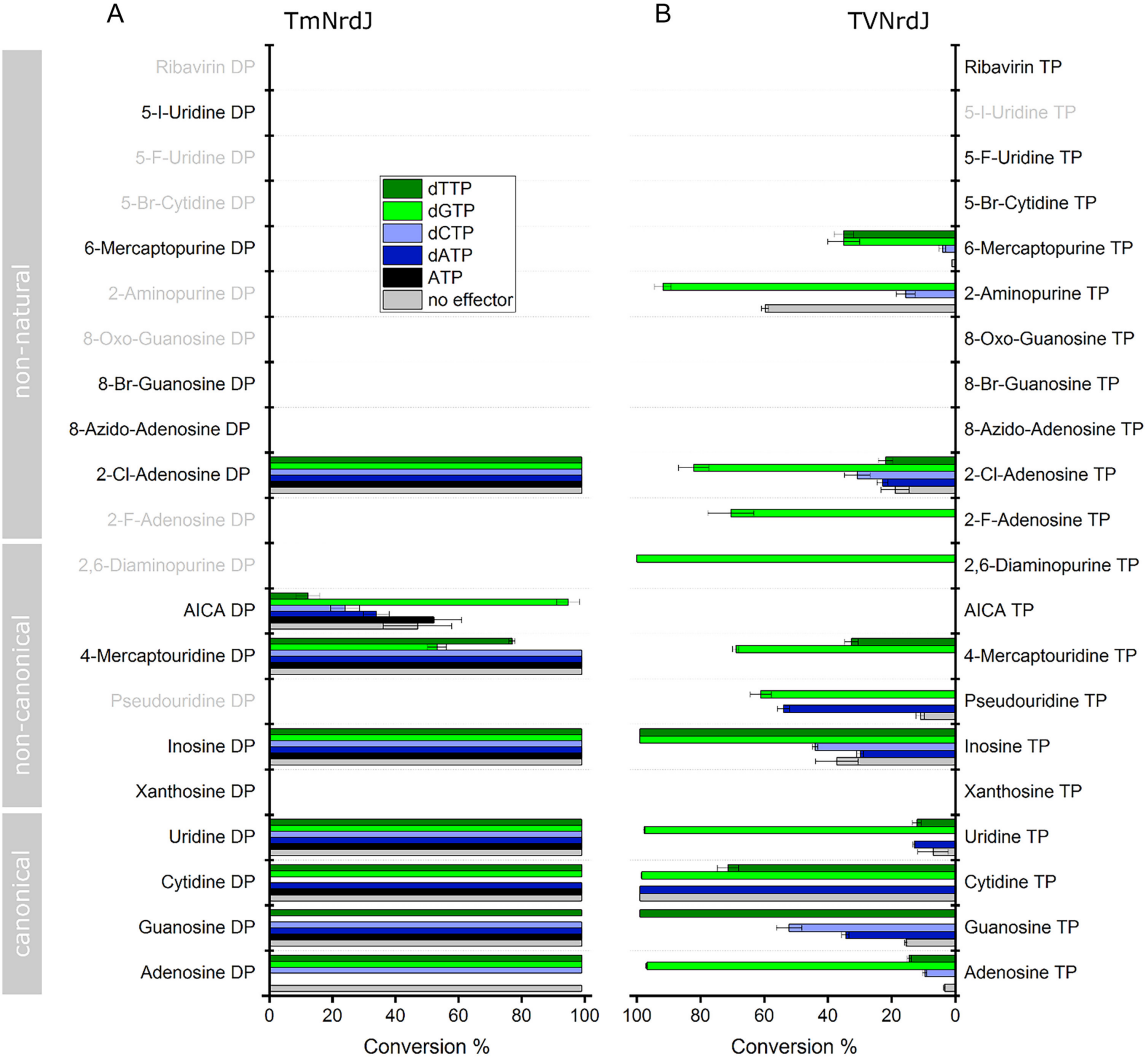
RNR substrate spectrum. (A) For the RNR from *T. maritima* TmNrdJ, the substrate spectrum was tested with a set of canonical, noncanonical nucleoside and non‐natural nucleoside diphosphate substrates. For each substrate, the conversion was tested with different allosteric effectors (dTTP, dGTP, dCTP, dATP, and ATP) and without effector. (B) For the RNR from the Thermus virus TVNrdJ, the substrate spectrum was tested with a set of canonical, noncanonical and non‐natural nucleoside triphosphate substrates. For each substrate, the conversion was tested with different allosteric effectors (dTTP, dGTP, dCTP, and dATP) and without effector. Grey nucleotides were not tested due to unavailability. The error bars indicate the standard deviation from three independent experiments.

The substrate specificity of both model RNRs was determined for a broad set of nucleotide compounds in three categories (Figure 3). The first are the canonical nucleotides, which are the constituents of natural RNA and DNA. The second are natural but noncanonical nucleotides, which appear in cellular metabolism but are not part of the canonical genetic code. The third are non‐natural noncanonical nucleotides, which do not naturally occur in cellular metabolism.

The TVNrdJ is a natural nucleoside triphosphate reductase, and was tested with the respective nucleoside triphosphates as substrates. In contrast, the TmNrdJ is a natural nucleoside diphosphate reductase, and was tested with the respective nucleoside diphosphates as far as they were available.

Another relevant aspect for RNR substrate specificity is the allosteric regulation. Since the presence of an effector molecule modifies the substrate specificity of RNRs, we tested each substrate in the presence and absence of effectors (dNTPs and ATP). For nucleoside triphosphate reductase TVNrdJ, ATP was omitted as effector, since it can serve as a substrate itself. In order to observe also lower activities, both enzymes were applied in high concentrations (4–5 µmol L^−1^). Both enzymes achieved full conversion for all four canonical substrates. For the TVNrdJ, full conversion was reached mainly in the presence of the cognate effector, with notable exception CTP as substrate. For the TmNrdJ, full conversion was achieved in the presence of all effectors and in the absence of any effector.

Both enzymes were also able to convert most of the tested natural noncanonical nucleotides. One notable exception was the xanthosine nucleotides XDP/XTP, which was not converted by both enzymes regardless of the effector. In contrast, the inosine nucleotides IDP/ITP was converted by both enzymes. For TVNrdJ, the conversion was driven by the presence of the effectors dTTP and dGTP, which commonly facilitate the activity for GTP and ATP, respectively. The 4‐thio uridine nucleotides were converted by both enzymes as well, however, with differing preferences in terms of the effector. For the AICA ribonucleotide and 2,6‐diaminopurine [36, 37], TmNrdJ showed activity with the highest observed conversion with the effector dGTP, the cognate effector of ATP. For the ribose modified pseudouridine triphosphate, TVNrdJ showed conversion with the effectors dATP and dGTP.

In terms of non‐natural nucleotides, several nucleotides with modifications on different positions of the purine or pyrimidine core structure were tested. Conversion was observed for 2‐F‐adenosine, 2‐Cl‐adenosine, 2‐aminopurine, and 6‐mercaptopurine nucleotides. For 2‐F‐adenosine triphosphate, 2‐Cl‐adenosine triphosphate, and 2,6‐diaminopurine, TVNrdJ showed high conversions with the effector dGTP. 2‐aminopurine triphosphate was converted either with dGTP or without any effector. As for 6‐mercaptopurine triphosphate, the highest conversion was observed with the effectors dTTP and dGTP. No activity was detected for purine nucleotides modified at position 8 and pyrimidine nucleotides modified at position 5.

### 2.3 Inosine Nucleotide Kinetics

As inosine nucleotides are present in the cellular context and could be problematic for the cell, the high conversions of IDP and ITP could be relevant from a physiological point of view. To evaluate this, we measured the kinetic parameters for both model enzymes for the canonical purine nucleotides. For ADP/ATP and GDP/GTP, the respective cognate effectors dGTP and dTTP, were applied. For IDP/ITP, both of these effectors were tested. Michaelis–Menten like behavior was observed for all tested substrate–effector pairs except for IDP/dTTP and ITP/dGTP (Table 1). *K*_M_ values for all successfully tested pairs were between 0.2 and 0.9 mmol L^−1^. Likewise, the *V*_max_ values were in the same order of magnitude, ranging between 133 and 424 mU mg^−1^.

**TABLE 1:** Kinetic parameters of the TmNrdJ and the TVNrdJ for the conversion different purine nucleotide with different effectors. Table entries without kinetic data indicate experiments where no Michaelis–Menten like behavior was observed. The indicated errors represent the standard error of the nonlinear regression.

Enzyme	Substrate	Effector	*V*_max_, mU mg^−1^	*K*_M_, mmol L^−1^
TmNrdJ	ADP	dGTP	237 ± 16	0.81 ± 0.4
	GDP	dCTP	424 ± 9	0.30 ± 0.02
	IDP	dGTP	133 ± 1	0.24 ± 0.02
	IDP	dTTP	—	—
TVNrdJ	ATP	dGTP	215 ± 1	0.42 ± 0.02
	GTP	dTTP	171 ± 1	0.25 ± 0.04
	ITP	dGTP	—	—
	ITP	dTTP	189 ± 15	0.90 ± 0.16

### 2.4 Implications for Nucleotide Metabolism

RNRs are described to play a major role in the regulation of the nucleotide metabolism, balancing the cellular dNTP pools through their intricate allosteric regulation [31]. The capability to convert the four canonical nucleotides necessitates the capability to convert noncanonical nucleotides, present in the cell. While XDP/XTP is excluded, IDP/ITP can be converted by both tested enzymes. The similarity of the inosine nucleotides to both adenosine and guanosine seems to make it impossible for the enzyme to distinguish between them, which is supported by the comparable kinetic parameters.

**FIGURE 4:**
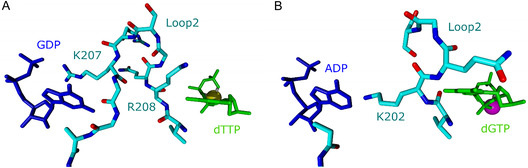
Active site and specificity site of TmNrdJ with bound substrate and effector. (A) The active site of the TmNrdJ with bound substrate GDP and the specificity site with bound effector dTTP (PDB: 1XJE). (B) The same active site with bound substrate ADP and the specificity site with bound effector dGTP (PDB: 1XJK). Loop2 lies between the active site and the specificity site, responsible for the regulatory effect [32].

The results generally support the mechanism of allosteric regulation as described previously [32]. The binding of guanosine nucleotides is supported by *π*–*π* stacking interactions between K207 and the extended *π* system of purine nucleotide with a 6‐oxo‐function (Figure 4A). Accordingly, purine nucleotides with 6‐oxo or 6‐sulfo groups (inosine, 6‐mercaptopurine) can be converted with the effector dTTP. The binding of adenosine nucleotides is supported by a hydrogen bridge between K202 and the 5´‐N‐position of adenosine (Figure 4B). This is also true for the noncanonical inosine, 2‐aminopurine and 2,6‐diaminopurine nucleotides.

The synthesis of dIDP and dITP seems to be an unavoidable consequence of the broad substrate scope of the RNRs, with possible consequences for the stability of the genetic information [38]. Inosine di‐ and triphosphate can be produced by nucleotide kinases as a byproduct of the synthesis of the canonical nucleotides [39, 40]. dITP and dXTP in turn, can be incorporated into DNA, resulting in mutation or double strand breaks if not repaired [40]. Our results show that the two tested RNRs are capable of the conversion inosine but not xanthosine nucleotides, indicating the possibility of a complete enzymatic dITP production from IMP. Cells solve this metabolic problem with ITPases, catalyzing the dephosphorylation of ITP, dITP as well as XTP [41, 42]. The described preference of these enzymes for inosine nucleotides is thereby consisted with the capability of RNRs to produce dIDP/dITP and the threat this poses for the genetic information.

### 2.5 Implications for Biocatalysis

With their capability to reduce ribonucleotides to the corresponding deoxyribonucleotides, RNRs are an important puzzle piece for the biosynthesis of noncanonical dNTPs, the natural substrate spectrum contains structurally divers purine and pyrimidine nucleotides [32, 34].

We tested a selection of noncanonical nucleotides, focusing mainly on modified natural nucleobases or close analogues. Generally, both tested enzymes are capable of converting most nucleotides with modifications at pre‐existing functional groups. Switching amino and oxo functions as well as replacing oxygen with sulfur seems to be acceptable in most cases. Also, the introduction of a fluorine or chlorine function in position 2 of the purine was accepted by both enzymes. However, nucleotides with functional groups at positions with no natural substituents, such as position 5 in pyrimidines or position 8 in purines, seem to be less acceptable. Nucleotides are bound in the active site of the enzyme in a conformation, where both the 5´‐position of pyrimidine bases and then 8´‐position of purine bases point towards the phosphate moiety of the nucleotide (Figure 4) [32]. Therefore, sterical hindrance within the substrate seems to prohibit those modified nucleotides to bind to the enzyme in a constructive manner.

Depending on the desired application for the non‐natural dNTP, only small modifications are necessary. NMR applications involve fluorinated nucleotides, which are accessible with RNRs at least in part (2‐F‐dATP) [15, 43]. The same is true for many candidate nucleotides for the augmentation of the genetic code, where single exchanges of oxo and amino functions are a preferred way to diversify the existing alphabet [44, 45, 46, 47]. Deoxy‐2,6‐diaminopurine nucleotides are one building block of the eight‐letter code hachimoji base pairs [46].

While other biocatalytic pathways for the production of deoxyribonucleotides have been published, they all require the provision of deoxyribose in one form or another and mainly rely on kinases [14, 18, 48]. The application of RNRs allows to exploit well‐established biocatalytical cascades for the biosynthesis of ribonucleotides to produce the corresponding deoxy‐ribonucleotide without external provision of deoxyribose. Since the RNR catalyzed reaction is a practically irreversible redox reaction, it can help in driving the whole cascade, thermodynamically. The main disadvantage of this cascade lies in the product being nucleotide mixtures (dNTP, NTP, dNDP, and NDP) caused by polyphosphate kinase thermodynamic limitation [49]. Other published cascades may provide higher purity NTPs and thus, higher purity dNTPs [14]. On the other hand, higher purity may not be necessary if the product dNTPs are used in situ, for instance for incorporation into DNA.

Although only class II RNRs were tested in this study, the overall similarity of the substrate spectrum and allosteric regulation may be similar in the other two RNR classes, class I in particular. The study shows that the tested enzymes are less accommodating for ribonucleotide substrates with larger modifications or modifications on specific positions of the nucleobase. Protein engineering may be the pathway forward to modify the nucleobase binding site. This may enable the synthesis of a broader variety of nucleotides and makes RNRs even more powerful biocatalysts as they currently are.

## 3 Conclusion

RNRs are a valuable tool for the biosynthesis of noncanonical deoxyribonucleotides. Due to their broad substrate spectrum, well‐established cascades for the production of ribonucleoside triphosphates can be extended to produce the corresponding deoxyribonucleotides. Understanding and improving the substrate spectrum of RNRs will also help in the creation of in vivo biosynthesis pathways for the biosynthesis of noncanonical nucleotides, which would open the door for other technologies, such as a true augmentation of the genetic code, all life is based on.

## 4 Experimental

If not stated differently, all the used chemicals were purchased from Sigma–Aldrich (Steinheim, Germany) or Carl Roth (Karlsruhe, Germany). Ribonucleotides and deoxyribonucleoside di‐ and triphosphates were purchased from Jena Bioscience (Jena, Germany).

### 4.1 Protein Production

Recombinant expression of the *nrdJm* from Thermus virus P74−26 (UniProt ID: A7XXH5) [34, 35] was carried out using a synthetic gene in a pET28b(+) plasmid with N‐terminal His tag. The *nrdJ* from *T. maritima* was cloned from genomic DNA (UniProt ID: O33839) [32]. For the expression of both enzymes, the *E. coli* BL21(DE3) strain was used. For each enzyme, LB media containing 30 µg mL^−1^ kanamycin was inoculated from overnight preculture with an initial OD_600_ of 0.1 and then incubated at 37°C and 130 rpm. As the cultures reached an OD_600_ of 1.0, expression was induced by IPTG addition to a final concentration of 0.1 mmol L^−1^. Both cultures were incubated at 37°C for 4 h and 130 rpm. The cells from cultures were harvested and then stored at −20°C. The cells were resuspended in DNAse containing wash buffer and (50 mmol L^−1^ tris, 300 mmol L^−1^ NaCl, 10 mmol L^−1^ imidazole, pH = 8.0) and treated with 5 mg ml^−1^ lysozyme for 45 min at 4°C. After sonication (4 times 1 min), the crude extract was centrifuged at 20.000 g for 45 min. The clear lysate was later loaded to the HisTrap FF crude 5 ml column (GE Healthcare, Chicago, IL, USA) and eluted in His‐elution buffer (50 mmol L^−1^ tris, 300 mmol L^−1^ NaCl, 500 mmol L^−1^ imidazole, pH = 8.0). The eluted proteins were desalted by HiPrepTM 26/10 desalting column (GE Healthcare, Chicago, Illinois, USA) in the desalting buffer (50 mmol L^−1^ tris, 300 mmol L^−1^ NaCl, pH = 8.0) to remove imidazole. The purified proteins were aliquoted and stored in 18% (w/v) glycerol at −80°C.

### 4.2 Ribonucleotide Reductase Activity Assay

To investigate the optimum temperature of the TmNrdJ, enzyme activity was tested at a temperature range of 30°C–95°C (Figure 2). Under standard conditions, assays for both enzymes were performed in a final volume of 50 µl at 60°C. The TV74 NrdJ reaction mixture consisted of 50 mmol L^−1^ tris buffer adjusted to pH = 8.0, 20 mmol L^−1^ MgCl_2_, 20 mmol L^−1^ DTT as reductant, 10 μmol L^−1^ Adenosylcobalamin (cofactor B12), 5 µmol L^−1^ enzyme, 0.5 mmol L^−1^ of the tested nucleoside triphosphate and 1 mmol L^−1^ of the effector. The same reaction mixture was applied for *T. maritima* NrdJ; however, the concentration of the enzyme was lowered to 4 µmol L^−1^, also Adenosylcobalamin (cofactor B12) to 8 µmol L^−1^, and 0.5 mmol L^−1^ of the tested nucleoside diphosphate. For six noncanonical nucleotides (ZDP. ZTP, 2‐Cl‐ADP, 2‐Cl‐ATP, 2‐F‐ATP, and 2,6‐Aminopurinetriphosphate) substrates were synthesized in situ via the an enzymatic cascade published previously [10, 50]. The cascades were performed with a starting concentration of 1 mmol L^−1^ of the respective nucleobase, yielding NDP/dNDP concentrations of 0.85 mmol L^−1^ and NTP/dNTP concentrations of 0.65 mmol L^−1^ in sum [10, 50]. The nucleoside diphosphate reductase was tested with the effectors ATP, dATP, dCTP, dGTP, and dTTP, with no effector. The nucleoside triphosphate reductase was tested with the same effectors except for ATP.

The reaction started with the addition of the enzyme to the respective reaction mixture and was incubated for 20 and 30 min for TmNrdJ and TV74NrdJm, respectively. The reaction was terminated by adding an equivalent amount of 100% methanol, then mixing vigorously and heating at 95°C for 15 min. The reaction mixture was diluted with four volumes of water and then centrifuged to remove the precipitated enzymes. The same assay was conducted to evaluate the kinetic parameters, with different concentrations of substrates and time intervals, with enzyme concentrations ranging from 0.5 to 1.6 µmol L^−1^. The Michaelis–Menten constant *K*_M_ and the maximum velocity *V*_max_ were estimated by nonlinear regression of the experimental data to the Michaelis–Menten equation (OriginPro 2019).

### 4.3 HPLC Analytics

The formed deoxyribonucleoside di‐ and triphosphates were quantified by injecting 5 µl sample on a reversed‐phase Eurosphere II 100‐5C18 column (Knauer, Berlin, Germany) connected to high‐performance liquid chromatography (HPLC) Knauer Azura‐HPLC (Knauer, Berlin, Germany) with several gradients of methanol to obtain maximum resolution between the nucleosides di‐and triphosphate as substrate and the corresponding deoxyribonucleoside di‐and triphosphates [51]. The measurements were calibrated using deoxyribonucleotides or, if unavailable, the ribonucleotides. The analytics were performed with a flow rate of 0.3 ml min^−1^ with the following buffers as mobile phase buffer A: 50 mmol L^−1^ KP*_i_*‐Buffer (pH = 7.0), 10 mmol L^−1^ tetrabutylammonium hydroxide (TBAH), and 10% (v/v) methanol; buffer B: 50 mmol L^−1^ KP*_i_*‐Buffer (pH = 7.0), 10 mmol L^−1^ TBAH, and 30% (v/v) methanol. Various gradients of methanol buffer were used to achieve maximum resolution, and details are given in the supporting information.

Detection was performed via UV absorption at *λ *= 260 nm for all nucleotides except for the sulphur containing ones. Here, a wavelength *λ *= 330 nm was applied. In general, peaks were identified with analytical standards. For those products, where the respective nucleotide was not commercially available, liquid chromatography‐mass spectrometry (LC‐MS) was applied for peak identification. The conversion data in Figure 3 were calculated by dividing the integrated area of the product peak by the sum of the area of the product and substrate peak as described previously [14].

### 4.4 LC‐MS Analytics

Samples were prepared by diluting the already used samples for the HPLC with acetonitrile 1:1 and measured by Thermo vanquish‐HPLC (Thermo Fisher Scientific, Waltham, MA, USA) with a ZIC‐cHILIC 3 µm, 100 Å column (Merck Millipore, Burlington, MA, USA). Separation was conducted at a flow rate of 0.3 mL min^−1^ with the following eluents: (A) 10 mmol L^−1^ ammonium acetate buffer (pH = 6.8) and 75% (v/v) acetonitrile; (B) 10 mmol L^−1^ ammonium acetate buffer (pH = 6.8) and 0% (v/v) acetonitrile. The following elution profile was used: 0 min 100% A, 10 min 70% A, 12 min 25% A, 13 min 25% A, 13.5 min 100% A, and 16 min 100% A.

Mass spectrometric analysis was performed on a coupled Thermo Q Exactive Mass spectrometer (Thermo Fisher Scientific, Waltham, MA, USA). The analysis was performed with the following scan parameters: Polarity: Negative or positive (Table S1), AGC target: 3 × 10^6^, maximum IT: 200 ms; scan range: 250–1000 *m*/*z*. Resolution 70,000. The electron spray settings were spray voltage: 4 kV; capillary temperature 320°C; sheath gas flow rate: 25 mL min^−1^; AUX gas flow rate: 10 mL min^−1^; S‐lens RF level: 55.

## Funding

This study was supported by Deutsche Forschungsgemeinschaft (LO2678/2‐1, INST269/780‐1FUGG).

## Conflicts of Interest

The authors declare no conflicts of interest.

## Supporting information

Experimental details concerning enzyme provision and analytics are given in the supporting information.

## Data Availability

The data that support the findings of this study are available in the supplementary material of this article.
