# Supplementary material for: Substrate Specificity of B12‐Depedent Ribonucleotide Reductases: Biotechnology and Metabolic Implications
Source: Chembiochem. 2026 Jun 15;27(11):e202500344. doi: 10.1002/cbic.202500344 (PMC13266583; doi:10.1002/cbic.202500344)
Supplement: Supplementary file 1 — Experimental details concerning enzyme provision and analytics are given in the supporting information. [file CBIC-27-e202500344-s001.pdf]

Supporting Information

Substrate Specificity of B12-Depedent Ribonucleotide Reductases: Biotechnology and Metabolic Implications

*Lobna Eltoukhy, and Christoph Loderer**

Chair of Molecular Biotechnology, Institute for Microbiology, Technische Universität Dresden, Zellescher Weg 20b, 01217 Dresden, Germany

KEYWORDS: ribonucleotide reductases, non-canonical deoxyribonucleotides, biocatalysis, allosteric regulation, non-natural nucleic acids.

1. SDS-PAGE of the purified enzymes

The two ribonucleotide reductases TmNrdJ and TVNrdJm from Class II were purified from *E. coli* cells after cell lysis using a HisTrap ™column installed in FPLC. The enzymes were eluted in a buffer containing imidazole. The imidazole was removed using a desalting column. The SDS-PAGE gel was prepared, and purified enzymes were loaded along with a protein ladder with a size range (10-250) kDa (**Figure S1**).

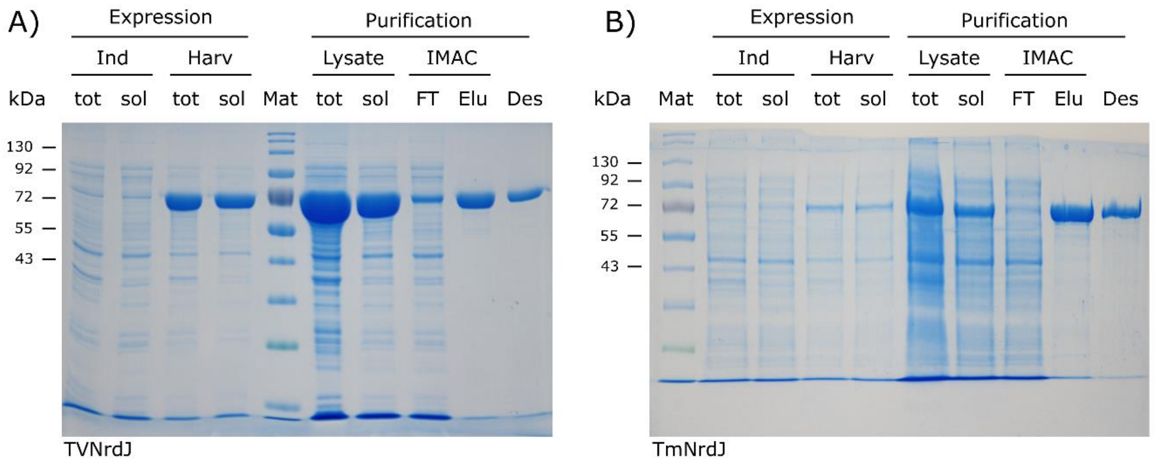

Figure S1. SDS-PAGE: Gene expression and purification of the model enzymes. The SDS-PAGEs for A) TVNrdJ and B) TmNrdJ show samples from gene expression, cell lysis and purification by IMAC and desalting. As standars, the color protein standart broad range (NEB) with a size range (10-250) was applied. The calculated molecular weights of the enzymes are 73 kDA for TVNrdJm and 70 kDa for TmNrdJ. Ind: sample at induction; Harv: sample at cell harvest: tot: total fraction; sol: soluble fraction; FT: flowthough of the IMAC; Elu: elution of the IMAC; Des: Desalting after IMAC.

The maino acid sequences are preseted below and contain an N-terminal His-tag from the pET28b(+) expression vector. The 6xHis-tag including a linker and a thrombin cleavage site are colored grey.

>TVNrdJm

MGSSHHHHHHSSGLVPRGSHMESLMNPLGRVVYLRTYSRFLEDKQRRETWPETVKRVVDYS
ASLAKVEEQEKLKLYDTFLHLRGFPAGRTLWAGGTPFIAQNGQANYNCAFTDLRTGKDFYD
LVILLMSGAGVGFRVTRDNIEALNQNLPIRRVPKLHVLPYEFYGYGHPLYTERSFIKRMGREA
TLVVGDSREGWAEAVKLFLELLGDRHVELLHVNLNSVRPLGSPLKRFGGFASGPGPLEDFFL
NAAFILGGTKPEGWTDVKALDVANLIGRMVVAGGTRRSAQISLGDWDSKNFIAAKTGNWW
ATAPWRAQSNNSVLFYAKPDRDTLLAFFDAVLQYGEPGFVNAQTALKRREDFRGVNPCSEIL
LRNKGVCNLTTVVLPNHVRNGKVDYALLEDTLRLLTRHAIRITTAQFPDVLSEWQRVQDEDR

LIGVSFTGLDDFIHLTGLDNDALAKVLAWMRSVVNDEAKRYSAELGLPKPKLATTVKPEGTL
SLLAGVSSGVHPAYAPYYIRRVRINKHDSVAQALRALGMEPKPEVGYDSLDTADVWVFEFPV
KTNAKRKAHDYTAVEQLERYKLVNSVYTEHNTSITVYVAPEEKEEVVDWLLQNWDHYVAV
SFLPKDDSAYPLMPFETISEEEYNALVAKLPDFSTLDEDVAFYDRLGGLMGDDIDPSCATGAC
PVR

>T.maritima NrdJ

MGSSHHHHHHSSGLVPRGSHMKLSDLISRWIDVEPSKNAQIILRDRYFMKDLDGNYLETKWE
DVARRVARVVATAELLNPSYKKNEKLDRIKEWEDIFFRVLKARLFIPNSPTLFNAGLGVKHDL
LWKPIDQMTLEDYEEIYRSRNHLHMLSACFVVPVGDSIEEIFEAVKEYALITKVGGGVGSNFS
ELRPKGSFVAGTHGKASGPVSFMHVFNSAISVVKQGYRRRGALMGILNINHPDIEEFIDAKKE
NTGEAVLNFFNLSVGFPMDKKEILKLYEEDGELELSHPRSTIRKKVKIRELFRKIATNAWKSG
DPGLAFLGEMNKYYPLYPHRKINSTNPCGEIGLSDYEACNLGSIDVAKFYNNGFVDLEALQEL
VQIAVRFLDNVIDVNVFPIDKITKAVKESRRLGLGIMGFADLLYKLEIPYNSQEARDFAANLM
AFIALHAHRTSYELGKEKGNFPLLEISRYRTEDNFVPFAMGMSNYDDEIREVMKMTKEFRRN
VALLTIAPTGSISNIADTSSGLEPNFLLAYTRFVTKEDGTKEPLLYVNQVLREKLNPEILKRIEK
ELIEKGSLKDIPDVPEKIKKVFVVALDIDPMDHLLMQDAFQRYVDNNISKTINMPQSATVDDV
LNVYLEALRTNVRGITVYRDGSLQTQVLTKALKTPEAPKVQFFVVDEKLKLHPRPRKDTLRS
VTRKYKRPDGTTYITISFDDTGEAVEIFISNGSEMAEAIGRLSSIALRAGVSIDEIVEQLSKVKGE
YCKGLAEEIKKALEDFAKLWLRTGEEAPESEEEPIEREKFIVAHNLRWQSGYYVDDEGNVYC
PVCLSKNSLIKQEGCVSCKNCGWSKCE

2. Quantitative analysis with HPLC

The analysis of the reduction of ribonucleotides was performed using a reversed-phase Eurosphere II 100-5C18 column (Knauer, Berlin, Germany) connected to HPLC Knauer Azura®-HPLC (Knauer, Berlin, Germany) with several gradients of methanol. The buffers were applied in the following elution profile: 0 min 100% A, 6 min 100 % A, 24 min 100 % B, 32 min 100% B, 33 min 100% A, and 36 min 100% A. The described elution profile was then further optimized to allow better separation and analysis of the formed deoxyribonucleotides di- and triphosphate.

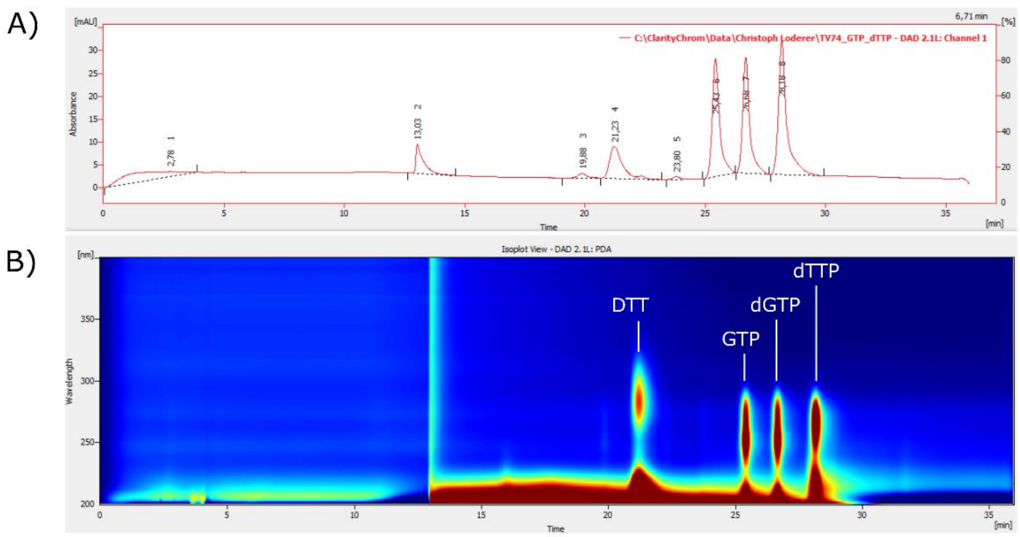

Figure S2. Representative HPLC chromatogram of the conversion of GTP with TVNrdJ: Shown are A) the UV trace at λ = 260 nm with a bandwith of 8 nm used for quantification and B) the spectrum from λ =200-400 nm, used for prak assignement.

<u>For the separation of IDP, ZDP, and ZTP</u>

The buffers were applied in the following elution profile: 0 min 100% A, 25 min 100% B, 33 min 100% B, 34 min 100% A, and 36 min 100% A.

<u>For the separation of ITP</u>

The buffers were applied in the following elution profile: 0 min 100% A, 27 min 100% B, 42 min 100% B, 43 min 60% A, and 46 min 60% A.

<u>For the separation of 2Cl-ADP and 2Cl-ATP</u>

The buffers were applied in the following elution profile: 0 min 60% A, 24 min 100% B, 33 min 100% B, 34 min 100% A, and 37 min 100% A.

3. Qualitative analysis using LC-MS

The same reactions measured by the HPLC were used to confirm the identity of the formed deoxyribonucleotides through their molecular weight. The mass peaks for each of the detected product compounds is shown in figure S3 with the expected and detected m/z values given in table 1.

Table 1:

Compund	Calculated m/z	Measured m/z
dIDP[-]	411.0191	411.0116
dITP[-]	490.9854	490.9781
ψ-dUTP[-]	468.9742	469.0071
4-Thio-dUDP[-]	402.9850	402.9780
4-Thio-dUTP[-]	482.9513	482.9219
dZDP[-]	403.0425	403.0410
2Cl-ADP[-]	445.9961	446.0034
2-Cl-ATP[-]	525.9624	525.9698
deoxy-2-aminopurine TP[+]	490.0013	489.9715
deoxy-6-mercaptopurine TP[+]	506.9626	506.9557

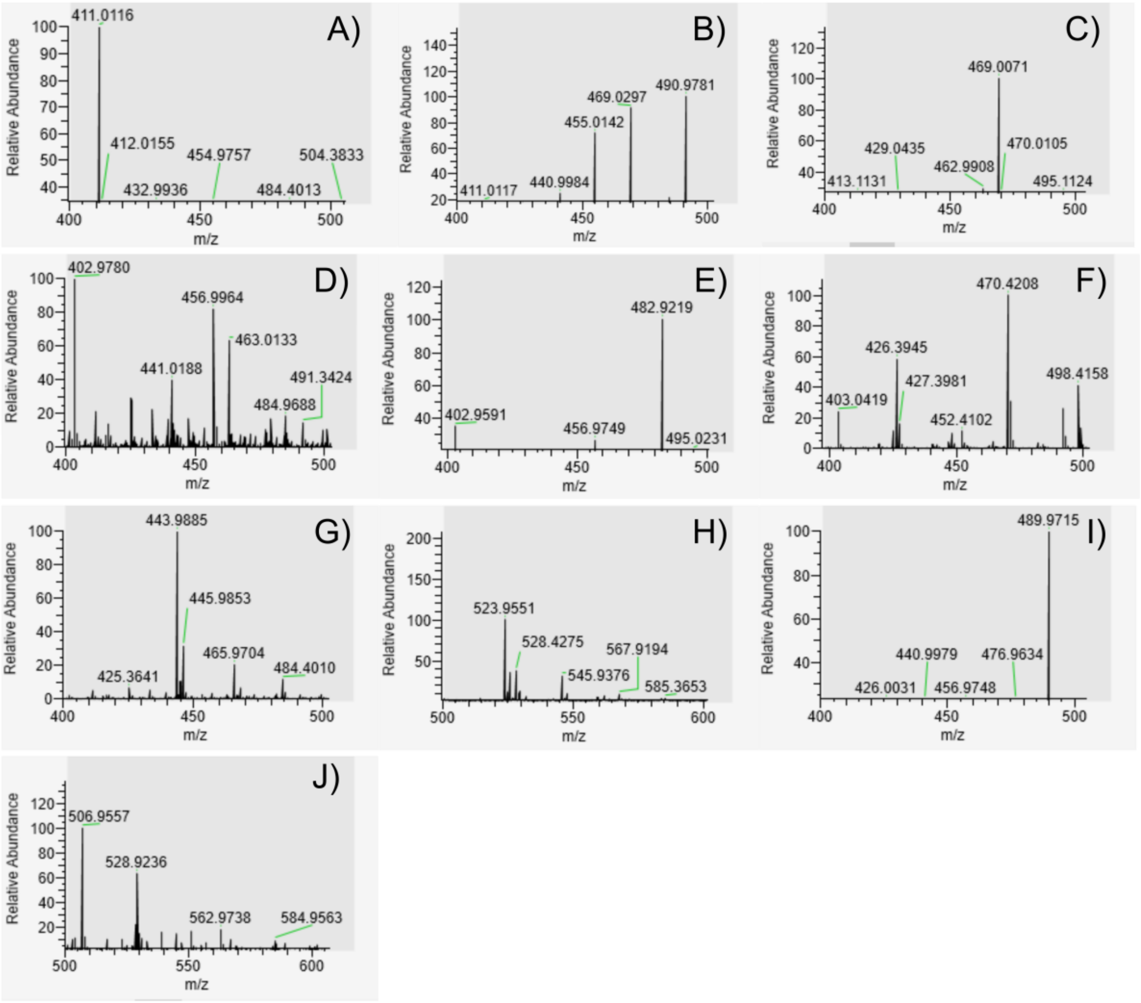

Figure S3. LC-MS identification of the deoxyribonucleotides. The mass peak was identified in the mass spectrum based on their molecular weight. A) dIDP, B) dITP, C) ψ-dUTP, D) 4-Thio-dUDP, E) 4-Thio-dUTP, F) dZDP, G) 2Cl-dADP, H) 2Cl-dATP, I) deoxy-2-aminopurine diphosphate, J) deoxy-6-mercaptopurine triphosphate.
